# Gut Microbiota Dynamics, Growth Performance, and Gut Morphology in Broiler Chickens Fed Diets Varying in Energy Density with or without Bacitracin Methylene Disalicylate (BMD)

**DOI:** 10.3390/microorganisms9040787

**Published:** 2021-04-09

**Authors:** Deborah Adewole, Fisayo Akinyemi

**Affiliations:** Department of Animal Science and Aquaculture, Faculty of Agriculture, Dalhousie University, Truro, NS B2N 5E3, Canada; fisayo.akinyemi@dal.ca

**Keywords:** bacitracin methylene disalicylate, energy density, gut microbiota, growth performance, broiler chickens

## Abstract

High-energy-density diet could increase body weight at the expense of the intestinal health of the animals. In order to optimize production without negatively influencing the gut health of chickens, dietary supplementation with bacitracin methylene disalicylate (BMD) is a common feeding strategy adopted to enhance production performance and intestinal health. Studies have suggested that BMD could improve chicken growth performance and gut health through modulation of the gut microbiota. The current study investigated the effect of BMD supplementation in a normal-energy (NE) or high-energy (HE) diet on growth performance, organ weights, jejunal morphology, and gut microbiota of broiler chickens at different growth stages. Birds were allocated to four treatments: normal-energy basal diet (NE-BAS), normal-energy BMD diet (NE-BMD), high-energy basal diet (HE-BAS), and high-energy BMD diet (HE-BMD). In the starter phase, body weight and body weight gain were reduced significantly (*p* < 0.05) in chickens fed HE diets compared to those fed NE diets. The FCR was significantly higher (*p* < 0.05) in birds fed HE-BMD diets in the starter phase but lower (*p* < 0.05) during the grower phase when compared to other treatments. Moreover, the relative bursa weight increased significantly (*p* = 0.0220) among birds that received HE diets. Birds fed HE-BMD had greater villus height (*p* = 0.054) than NE-BMD group. Among the chickens fed the HE diets, those that received BMD treatment had a significantly increased (*p* = 0.003) villus width (13.3% increase) compared to those that received the basal diet. Improved population of Firmicutes was observed in chickens fed HE-BMD diet when compared to HE-BAS. Our results imply that BMD may be more effective in improving intestinal health when supplemented in a high-energy diet for broiler chickens.

## 1. Introduction

Dietary energy density can be referred to as the amount of available energy per unit weight [1]. In the poultry industry, dietary energy and nutrient density have been reported to have a significant impact on gut health and growth performance [2,3,4]. The impact of high-energy-density diets in poultry could be beneficial, and at the same time detrimental. High-energy-density diets have been shown to increase body weight gain [5,6] and improve feed conversion efficiency [7,8] in poultry. Lamot et al. [9] reported an increase in gain-to-feed ratio during the first week of life for broiler chickens exposed to higher diet densities. However, aside from weight gain, the intestinal health of poultry birds is equally important as this can affect the farmer’s cost of maintaining a healthy flock. Studies have shown that changes in dietary energy density induce rapid changes in the composition of bacteria that colonizes the intestinal tract of mammals [10]. Other studies with mice and rats have shown that high-energy diets can trigger microbiota dysbiosis due to an imbalance between energy intake and expenditure [11,12,13]. A recent study with Pekin ducklings revealed that dietary energy content altered microbiota composition and diversity in the cecum [14]. 

In order to optimize production without negatively influencing the gut health of chickens, dietary supplementation with bacitracin methylene disalicylate (BMD) is a common feeding strategy adopted to enhance production performance and intestinal health. In Canada, BMD is regarded as an antibiotic of medium importance (that is, it has alternatives available) [15]. Although there are plans to eliminate the preventive use of BMD because of the worldwide concern about antibiotic resistance and the quest to preserve the potency of antibiotics for human and animal health [16], BMD is still currently being used in chicken production as a preventive measure against diseases [15]. BMD increases body weight gain and feed efficiency and promotes the cecal microbiota diversity in several poultry species [17,18,19]. Broiler chickens fed BMD showed a reduction in the concentrations of coliforms and *Lactobacillus salivarius* in the lower part of the intestine, along with significant growth-promoting effects [20]. 

It is plausible that bacitracin coupled with a diet of high or low energy density may elicit beneficial effects on the intestinal health of chickens. While various studies have focused on the effects of BMD on intestinal microbiota and growth performance of chickens [20,21,22,23], research on the effect of energy density and its interaction with the use of antibiotics in broiler chickens is limited. Therefore, it is important to give attention to dietary energy, how it affects gut microbiota in relation to antibiotic usage, and its influence on the growth performance of chickens. We hypothesized that BMD would complement the shortcomings resulting from feeding chickens with either low or high energy alone. In the present study, we determined the effects of BMD, two levels of dietary energy, and their interactions on the growth performance, organ weights, jejunal morphology, and gut microbiota of broiler chickens at different growth stages. 

## 2. Materials and Methods 

### 2.1. Management and Housing

Animal care approval for the experimental methods was granted by Dalhousie University Animal Use and Care Committee, and all chickens were handled and cared for according to the recommendations of the Canadian Council of Animal Care [24]. Broiler chicks (Ross 308) obtained at 1 day old from a commercial source arrived at the Atlantic Poultry Research Center, Dalhousie University Faculty of Agriculture, Truro, NS, and chicks were weighed in groups of 25 birds. Each group was assigned to a floor pen (0.93 m × 2.14 m), at a stocking density of 0.076 m^2^/bird. Room temperature was monitored daily and was gradually reduced from 31 to 22.6 °C from day 0 to day 42. The lighting program was set to produce 18 h of light and 6 h of darkness throughout the experimental period, and illumination was gradually reduced from 20 lx on day 0 to 5 lx.

### 2.2. Experimental Diets

Chickens received feed and water ad libitum via a phase-feeding program, which consisted of starter (0–14 days), grower (15–24 days), and finisher (25–42 days). Diets were fed as crumbled pellets during the starter phase and as pellets during the grower and finisher phases. Birds were randomly sorted into four dietary treatments, consisting of eight replicate pens per treatment. The experiment was designed as a 2 × 2 factorial design consisting of two levels of energy: (1) normal energy (NE) density and (2) high energy (HE) density and two levels of BMD inclusion: (1) a basal diet containing 0% of BMD (Basal) and (2) Basal + 0.05% BMD. The HE diets were formulated to contain an additional 100 kcal/kg ME above the NE diets, similar to the study of Kindlein et al. [25]. The NE and HE diets were formulated to have a similar ratio of metabolizable energy (ME) to crude protein and digestible amino acids, as presented in Table 1.

### 2.3. Data and Sample Collection

Feed intake (FI), body weight (BW), and mortality were recorded weekly, and body weight gain (BWG) and feed conversion ratio (FCR) were calculated. On days 21, 36, and 42 of age, one chicken per pen (eight chickens per treatment) was randomly selected and euthanized by electrical stunning and exsanguination. Birds were bled for 3–5 min after carotid and jugular veins were cut. After slaughter, digesta from the pair of ceca were mixed, sampled, and stored in plastic RNAse- and DNAse-free tubes, placed in liquid nitrogen, and afterward kept at −80 °C until ready for DNA extraction and sequencing. On day 42, visceral organs, including spleen, ceca, liver, bursa of Fabricius, and heart, were harvested and weighed by trained personnel. Samples from the jejunum (1.5 cm length midway between the point of entry of the bile ducts and Meckel’s diverticulum) were collected and fixed in 10% neutral buffered saline for histomorphological processing. 

### 2.4. Analysis of Diets and Histological Samples

#### 2.4.1. Diet Analysis

Nitrogen content in the diets was determined using the combustion method [26], Method 990.03, with a nitrogen analyzer (Model Leco CN828 Carbon Nitrogen Determinator, St. Joseph, MO, USA) and CP was calculated as N × 6.25. The ether extract in samples was determined after hexane extraction [26] using Method 920.39 in an Ankom XT10 Fat Extractor system (Macedon, NY, USA). The metabolizable energy content of diets was determined at the Central Testing Laboratory, Winnipeg, MB, using their standard laboratory procedures. 

#### 2.4.2. Histological Measurements 

Fixed jejunum samples were processed using the same procedure as Oladokun et al. [24]. Briefly, villus height (from the base of the intestinal mucosa to the tip of the villus excluding the intestinal crypt), villus width (halfway between the base and the tip), and crypt depth (from the base upward to the region of transition between the crypt and villi) were determined by taking approximately 10 measurements of each component per slide using an image processing and analysis system (Leica DC480; Leica Microsystems Imaging Solutions Ltd., Concord, ON, Canada).

### 2.5. DNA Extraction and 16S rRNA Gene Sequencing

Specimens were placed into a MoBio PowerMag Soil DNA Isolation aBead Plate. DNA was extracted following MoBio’s instructions on a KingFisher robot. Bacterial 16S rRNA genes were PCR-amplified with dual-barcoded primers targeting the V4 region (515F 5′-GTGCCAGCMGCCGCGGTAA-3′, and 806R 5′-GGACTACHVGGGTWTCTAAT-3′), as per the protocol of Kozich et al. [27]. Amplicons were sequenced with an Illumina MiSeq using the 300-bp paired-end kit (v.3). Sequences were denoised, taxonomically classified using Silva (v. 138) as the reference database, and clustered into 97%-similarity operational taxonomic units (OTUs) with the mothur software package (v. 1.44.1) [28], following the recommended procedure (https://www.mothur.org/wiki/MiSeq_SOP; accessed on 11 October 2020). The potential for contamination was addressed by co-sequencing DNA amplified from specimens and from template-free controls (negative control) and processing the extraction kit reagents the same way as the specimens. A positive control from “S00Z1-” samples consisting of cloned SUP05 DNA was also included. Operational taxonomic units were considered putative contaminants (and were removed) if their mean abundance in controls reached or exceeded 25% of their mean abundance in specimens.

### 2.6. Bioinformatics and Statistical Analyses

We sequenced 16Sv4 amplicons generated from cecal digesta samples on a MiSeq. MiSeq-generated Fastq files were quality-filtered and clustered into 97% similarity operational taxonomic unit (OTUs) using the mothur software package (http://www.mothur.org; accessed on 11 October 2020). The resulting dataset had 94,509 OTUs (including singletons). An average of 42,742 quality-filtered reads was generated per sample. Sequencing quality for R1 and R2 was determined using FastQC 0.11.5 (Supplementary Figures S1 and S2). The Shannon index was used to estimate alpha diversity [29] on raw OTU abundance tables after filtering out contaminants. The significance of diversity differences was tested with ANOVA or linear mixed model depending on the study design. To estimate beta diversity across samples, we excluded OTUs occurring with a count of less than 3 in at least 10% of the samples and then computed Bray–Curtis indices. Abundance-weighted sample pairwise differences were calculated using the Bray–Curtis dissimilarity. Bray–Curtis dissimilarity is calculated by the ratio of the summed absolute differences in counts to the sum of abundances in the two samples [30]. Beta diversity was visualized using principal coordinate analysis (PCoA) ordination, emphasizing differences across samples. Permutational multivariate analyses of variance (PERMANOVA) was used to assess variation in community structure, with treatment group as the main fixed factor, and 9999 permutations were used for significance testing [31]. We conducted all analyses in the R environment. DESeq2 R package was used to identify differentially abundant taxa among diet variables. The 2-sided Welch’s *t*-test [32] and Benjamini–Hochberg false discovery rate correction were adopted for the group analysis. We used STAMP software [33] to analyze the significant microbes among the treatments and different age groups. The principal component analysis (PCA) analysis of the microbial community relationship was also performed with STAMP software using ANOVA as statistical test (*p*-value < 0.05) and Tukey–Kramer as post hoc test. Further statistical analyses were performed using GraphPad prism Program (version 7.0.1). *p*-values < 0.05 were regarded as significant values. Results are presented as means ± standard deviation (SD). LEfSe was performed (http://huttenhower.sph.harvard.edu/galaxy; accessed on 20 November 2021) by using the relative abundance of genus to identify different taxa microbes between the time points and different diet groups. We only showed the differential features using the default parameters (LDA Score > 2, *p* < 0.05) [34].

The growth performance, jejunal morphology, and organ weights data were subjected to analysis of variance using the general linear models (GLM) procedure of SAS with energy level, BMD, and energy × BMD interaction as factors and the following parameters as variables: feed intake, body weight, body weight gain, feed conversion ratio, organ weights, villus heights, villus width, crypt depth, and ratio of villus height to crypt depth. *p*-values less than 0.05 were considered significant.

## 3. Results

### 3.1. Growth Performance, Organ Weight, and Jejunal Morphology

The effects of BMD and energy level on the growth performance of broiler chickens are shown in Table 2. There were no significant effects of dietary treatments on overall FI, BW, BWG, and FCR. However, during the starter phase, chickens fed BMD in the high-energy group had lower BW and BWG and higher FCR compared to those fed the basal diet in the normal-energy group, while other treatments were intermediate. During the starter phase, chickens in the high-energy BMD group had a higher FCR compared to those fed the basal diet in the normal-energy group. However, this was compensated for by a lower FCR during the grower phase compared to the other treatments. As illustrated in Table 3, bursa’s relative weight significantly increased (*p* = 0.0220) among the birds that received HE diets compared to those that received NE diets. There were no effects of BMD and energy level on the relative weights of the remaining organs. As presented in Table 4, birds fed HE-BMD had greater villus height (*p* = 0.054) than those provided with NE-BMD. Among the chicken birds fed the HE diets, those that received BMD treatment had a significantly increased (*p* = 0.003) villus width (13.3% increase) compared to those that received the basal diet. There were no effects of dietary treatment on crypt depth and VH/CD.

### 3.2. Composition of Chicken Gut Microbiota of Chickens Fed with or without BMD at Varying Energy Levels

**Figure 1:**
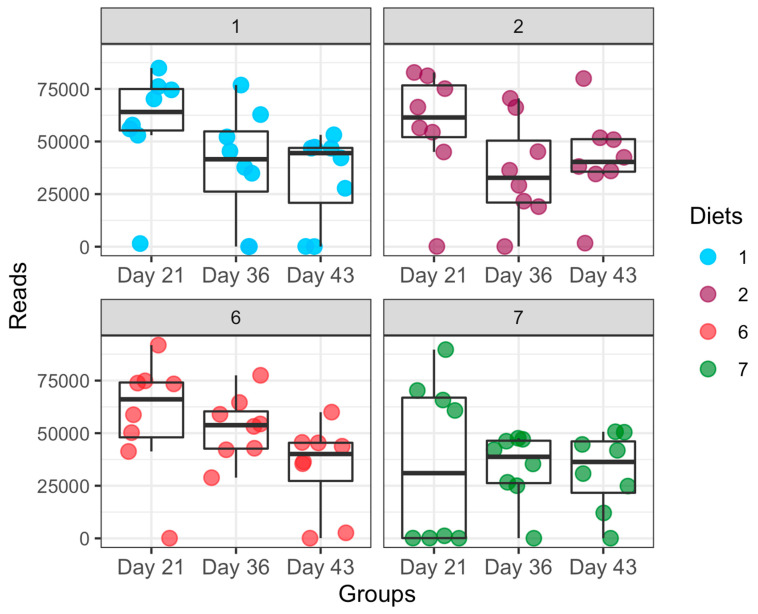
The total number of quality-filtered reads per sample. These reads reflect the total number of high-quality sequences that align with 16Sv4, clustered into OTUs, and were assigned taxonomic classification. Diets 1, 2, 6, and 7 represent normal-energy basal diet (NE-BAS), normal-energy diet with bacitracin methylene disalicylate (NE-BMD), high-energy basal diet (HE-BAS), and high-energy diet with bacitracin methylene disalicylate (HE-BMD), respectively.

A total of 94,509 OTUs were obtained from all samples. An average of 42,742 quality-filtered reads was generated per sample (Figure 1).

**Figure 2:**
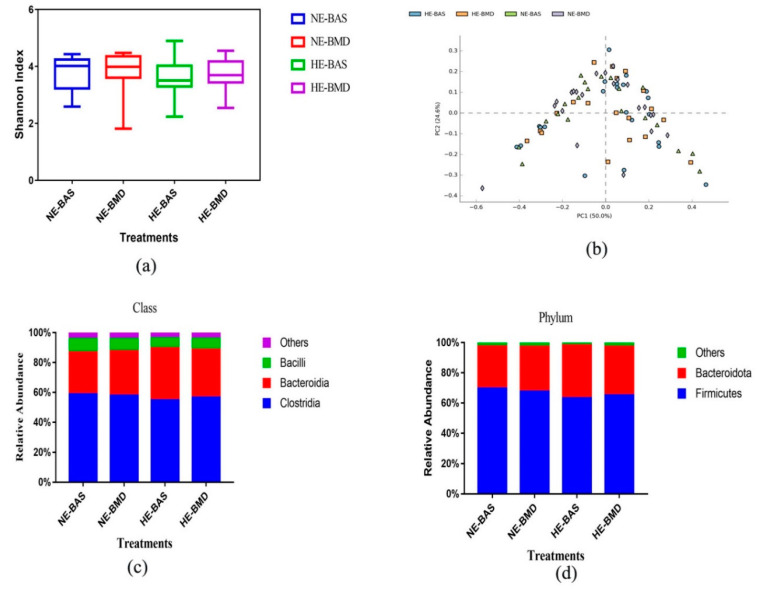

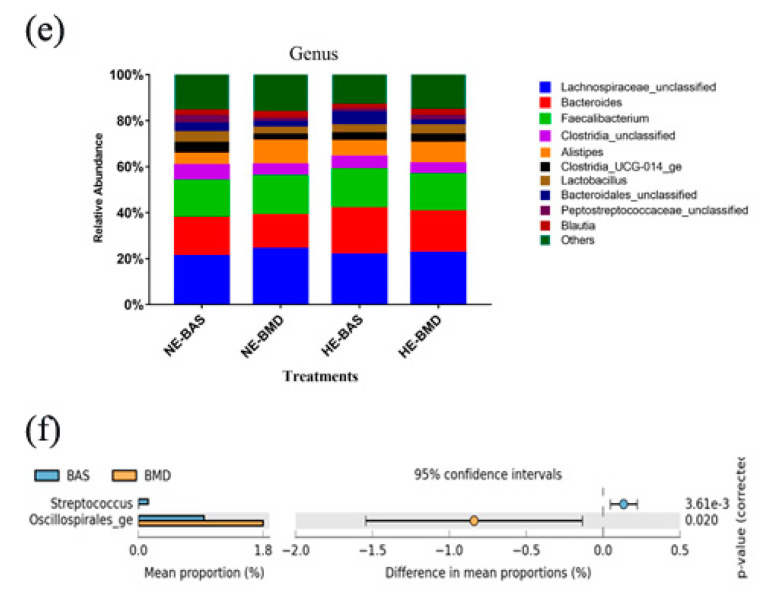
(**a**) Alpha analysis using the Shannon index showing the diversity of gut microbiota among the treatments (NE-BAS, NE-BMD, HE-BAS, and HE-BMD); the significance was tested with ANOVA and plot was generated using GraphPad prism. (**b**) The principal component analysis (PCA) plot, created using STAMP software, compares genus-level taxonomic profiles among different diets. NE-BAS = normal-energy basal diet; NE-BMD = normal-energy diet with bacitracin methylene disalicylate; HE-BAS = high-energy basal diet; HE-BMD = high-energy diet with bacitracin methylene disalicylate. (**c**) Relative abundance of top-class in broiler chickens fed different diets. (**d**) Relative abundance of top phyla in broiler chickens fed different diets. NE-BAS = normal-energy basal diet; NE-BMD = normal-energy diet containing bacitracin methylene disalicylate; HE-BAS = high-energy basal diet; HE-BMD = high-energy diet containing bacitracin methylene disalicylate. (**e**) Relative abundance of top genera in broiler chickens fed different diets (NE-BAS, NE-BMD, HE-BAS, and HE-BMD). Different colors represent different genera. (**f**) Significant differences in genera between the chickens fed basal (BAS) and bacitracin methylene disalicylate (BMD), assessed using STAMP software package (*p* < 0.05).

In the current study, we examined the effects of BMD on the chicken gut bacterial community at different energy levels. Alpha diversity analysis using the Shannon index, which accounts for richness and evenness, showed no differences among the four treatments, indicating that BMD did not influence microbiome evenness at different energy levels (Figure 2a). The PCA analysis revealed a weak influence of the treatments on gut microbial composition in the sampled chickens. A high level of similarity was observed among the treatments (Figure 2b). Additionally, we assessed the relative abundance among all the treatment groups at different taxonomic levels. The top classes were Clostridia, Bacteriodia, and Bacilli. Clostridia emerged as the most abundant in chickens fed NE, with a proportion of 58.9 and 58.0% for basal and BMD diets, respectively. Bacteriodia emerged as the most abundant in chickens fed high-energy diets, with a proportion of 34.8 and 32.0% for basal and BMD diets, respectively. Bacilli were most dominant in chickens fed NE (8.99 and 8.09% for basal and BMD groups, respectively) (Figure 2c). At the phylum level, the top two phyla among all treatments were Firmicutes and Bacteroidota. Firmicutes were more dominant in chickens fed NE diets, followed by those fed HE diets. The relative abundance was 69.6, 67.7, 63.3, and 65.2% for NE-BAS, NE-BMD, HE-BAS, and HE-BMD, respectively. Bacteroidota was seen as the most abundant in chickens fed HE diets, which include HE-BAS (34.77%) and HE-BMD (32.04%). The lowest proportion of Bacteroidota (27.95%) was observed among the chickens fed NE-BAS, while 29.72% was seen in NE-BMD (Figure 2d).

At the genus level, the dominant genera among the chickens fed with NE-BAS, NE-BMD, HE-BAS, and HE-BMD were *Lachnospiraceae_unclassified*, *Bacteroides*, *Faecalibacterium*, *Clostridia_unclassified*, *Alistipes*, *Clostridia_UCG_014_ge*, *Lactobacillus*, *Bacterodales*, *Peptostreptococcacea_unclassified*, and *Blautia*. The most dominant genus among chickens fed NE-BMD was *Lachnospiraceae_unclassified* (24.08%), while *Bacteroides* was the most abundant among chickens fed HE-BAS (20.11%). Irrespective of dietary energy density, we observed almost the same proportion of *Faecalibacterium* among chickens that received BMD or BAS (17.1 and 17.0%, respectively). *Alistipes* was most dominant in chickens fed BMD diets, irrespective of energy density. Chickens fed NE-BAS had the lowest proportion of *Alistipes* and the highest proportion of *Lactobacillus*, while those fed NE-BMD had the lowest abundance of *Lactobacillus* (Figure 2e). Two out of 83 genera were significantly different in abundance between the chickens fed BAS and BMD. *Streptococcus* was significantly abundant in chickens fed with BAS, while BMD inclusion triggered the drastic reduction of this microbe. *Oscillospirales_ge* was found to be differentially abundant in the chickens that received BMD treatment (Figure 2f). Moreover, we observed a reduction in the relative abundance of some intestinal bacteria in the BMD treatment group.

We identified 15 OTUs that were differentially abundant amongst treatments (NE-BAS, NE-BMD, HE-BAS, and HE-BMD) (FDR < 0.05), namely Otu00013, Otu00008, Otu00210, Otu00016, Otu00081, Otu00004, Otu00309, Otu00004, Otu00309, Otu00209, Otu00001, Otu00039, Otu00280, Otu00071, Otu00079, Otu00229, and Otu00150, which belong to the two phyla Firmicutes and Bacteroidota (Table 5).

**Figure 3:**
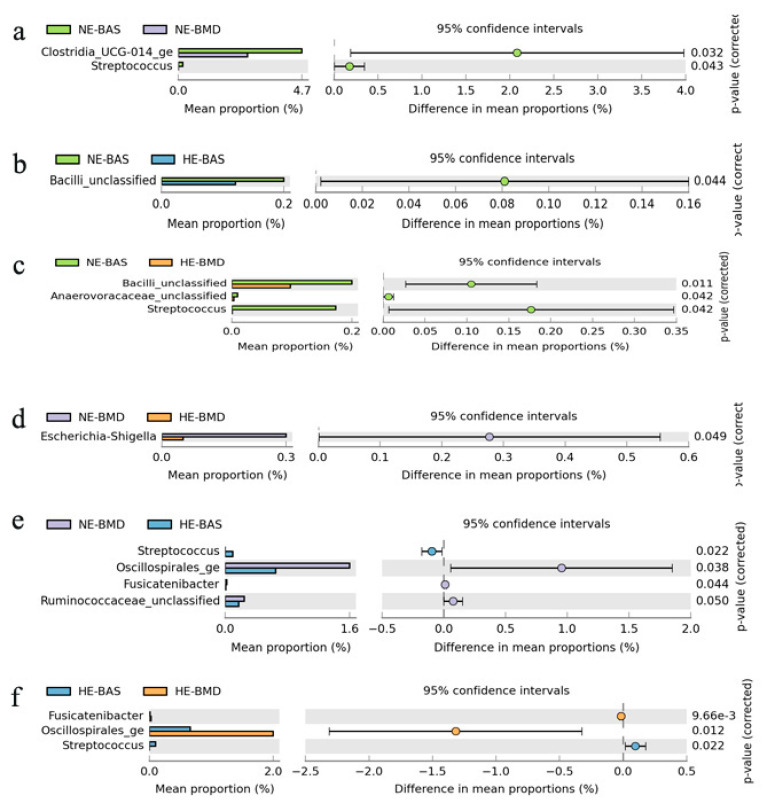
Significant differences in microbes among different diets using STAMP software package (*p*-value < 0.05). (**a**) Significant differences in microbes between chickens fed NE-BAS and NE-BMD. (**b**) Significant differences in microbes between chickens fed NE-BAS and HE-BAS. (**c**) Significant differences in microbes between chickens fed NE-BAS and HE-BMD. (**d**) Significant differences in microbes between chickens fed NE-BMD and HE-BMD. (**e**) Significant differences in microbes between chickens fed NE-BMD and HE-BAS. (**f**) Significant differences in microbes between chickens fed HE-BAS and HE-BMD. NE-BAS = normal-energy basal diet; NE-BMD = normal-energy diet containing bacitracin methylene disalicylate; HE-BAS = high-energy basal diet; HE-BMD = high-energy diet containing bacitracin methylene disalicylate.

Six phyla and 52 genera were observed in the broiler chickens. Further analysis using *t*-test was performed to evaluate the significantly abundant genera. Among the NE group, *Clostridia_UCG_014* was significantly different in abundance between chickens fed with basal and BMD diets. *Streptococcus*, *Oscillospirales*, *Fusicatenibacter*, and *Ruminococcacea_unclassified* were significantly different in abundance between chickens fed NE-BMD and HE-BAS. Among chickens fed HE, *Streptococcus*, *Oscillospirales*, and *Fusicatenibacter* were differential between basal and BMD diets. A higher abundance of *Escherichia–Shigella* was found to be significant in chickens fed NE-BMD diet. *Bacilli_unclassified* was significantly enriched (*p* < 0.05) in chickens fed NE-BAS (Figure 3). 

### 3.3. Dynamic Changes in Microbial Taxa across Growth Stages

**Figure 4:**
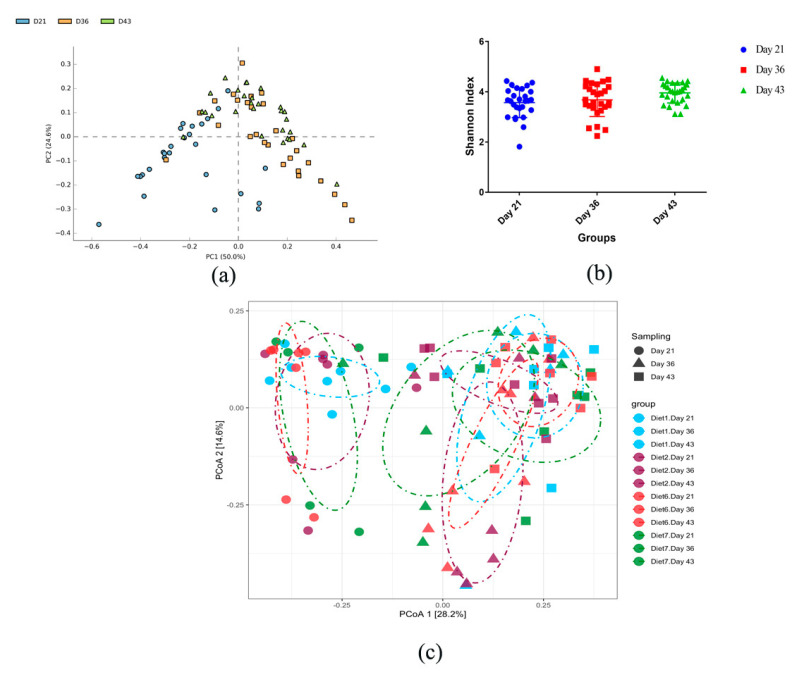

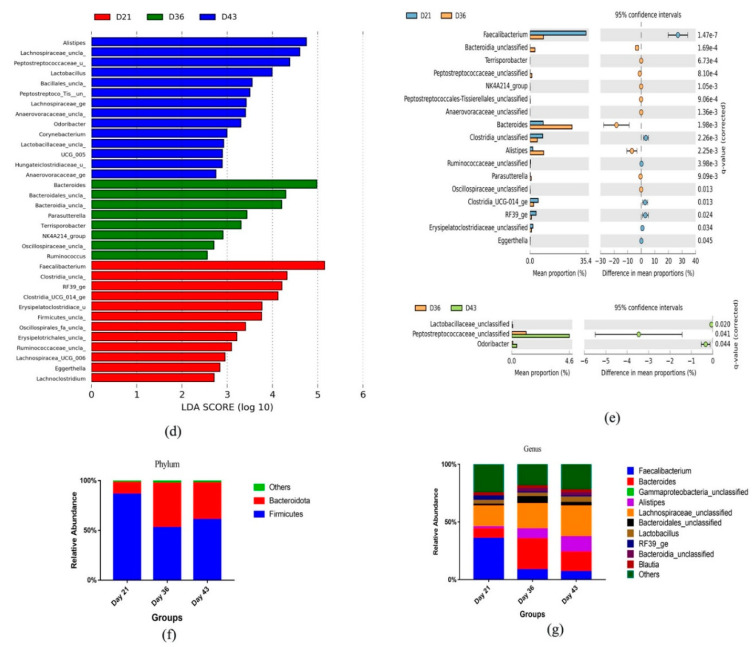
(**a**) The principal component analysis (PCA) plot, created using STAMP software, compares genus-level taxonomic profiles across growth stages (day 21 (D21), day 36 (D36), and day 43 (D41)). (**b**) Alpha analysis using the Shannon index, showing the diversity of gut microbiota at different growth stages (D21, D36, and D41). (**c**) Principal coordinate analysis (PCoA) plot obtained from beta analysis shows differences in bacterial community regardless of time. (**d**) LEfSe analysis result revealed the effect size of some significant taxa at different stages of growth. The plot was generated using the online LEfSe project. Red, green, and blue represent the enriched taxa in chickens on days 21, 36, and 43, respectively. (**e**) Significant differences in microbes between days 21 and 36 and between days 36 and 43 (*q*-value < 0.05), assessed using STAMP software package. (**f**) Relative abundance of top phyla during different stages of growth (days). (**g**) Relative abundance of genera during different stages of growth (days). Bar plots were generated using GraphPad Prism.

The principal component analysis (PCA) analysis showed significant differences across growth stages. We observed a high level of similarities between days 36 and 43 compared to day 21 (Figure 4a). Alpha diversity index using Shannon revealed a significant difference between days 21 and 43 and between days 36 and 43 (*p* < 0.05), although the difference between days 21 and 36 was not significant (Figure 4b). Moreover, the PCoA plot obtained from beta analysis showed that the interaction between diets and age influenced the microbial community of the broiler chickens. Greater similarity among diets on days 36 and 43 compared to day 21 was observed. Moreover, samples from different diets on day 21 clustered closer than those on days 36 and 43 (Figure 4c).

From the permutational analysis of variance (PERMANOVA) that was conducted to determine significant differences in beta diversity among sampling factors (Supplementary Table S1), we observed there were no significant differences in bacteria composition along with the treatments. Further post hoc pairwise testing indicated that there was a significant difference (*p*-value < 0.05) in gut microbial flora across the treatments at different days (Supplementary Table S2), which can be visualized by the above PCoA plot (Figure 4c). Pairwise contrasts are presented in Supplementary Table S2, and the FDR method was used to correct *p*-values for multiple comparisons.

LEfSe analysis result revealed the effect size of some significant taxa on different days (Figure 4d). Additionally, Welch’s *t*-test using Stamp software displayed the relative abundance of significant microbes among days. Specifically, 17 genera changed significantly between days 21 and 36, while 3 changed between days 36 and 43 (*q*-value < 0.05). Some of the genera that changed significantly were *Alistipes*, *Odoribacter*, *Bacillales_unclassified*, *Hungateiclostridiaceae_unclassified*, *Corynebacterium*, and *Parasutterella* (Figure 4e).

We further compared the abundance of gut microbiota in the broiler chickens at different growth stages. The most abundant phyla in all three time points were Firmicutes and Bacteroidota. On day 21, the proportion of Firmicutes was 86.3%, and the proportion of this phylum was reduced drastically to 52.8% and then increased to 61.0% on day 43. The highest proportion of Bacteroidota (44.49%) was seen on day 36, and the proportion of this phylum was later reduced to 36.6% on day 43. The lowest proportion of Bacteroidota (11.7%) was observed on day 21 (Figure 4f). A similar occurrence was seen at the genus level. The most abundant genera were *Faecalibacterium*, *Lachnospiraceae_unclassified*, *Bacteroides*, *Alistipes*, and *Clostridia_unclassified*. On day 21, the proportion of *Faecalibacterium* was 35.4%, and this genus also emerged as the most abundant. A minimal proportion of *Faecalibacterium* was observed on days 36 and 43 (8.45 and 6.75%, respectively). On day 36, *Bacteroides* emerged as the most abundant with a proportion of 26.6%, followed by *Lachnospiraceae_unclassified* (21.8%). On day 43, *Lachnospiraceae_unclassified* was the most dominant (26.6%). Notably, the relative abundance of *Lachnospiraceae_unclassified* increased with growth in broiler chickens (18.1, 21.8, and 26.5% on days 21, 36, and 46, respectively). Moreover, the relative abundance of *Alistipes* increased as broiler chickens increased with age. A low proportion of *Alistipes* (1.77%) was seen on day 21, and the proportion of this genus increased to 8.67% on day 36 and 13.50% on day 43. On the contrary, the relative abundance of *Faecalibacterium* decreased with growth changes (Figure 4g).

## 4. Discussion

### 4.1. Effect of BMD and Energy Density on Growth Performance, Organ Weight, and Jejunal Morphology

High-energy diet has been reported to improve BW, BWG [35,36], and FCR [37] of broiler chickens. In the current study, there was no significant effect of dietary energy on overall growth performance. There was an increase in FCR among the chickens fed high-energy diets during the starter phase, but this was later compensated for during the grower and finisher phases. Our result supports a previous study by Coon et al. [38] who found that chick weight gain for 0–28 days was not significantly different for chicks fed high-energy diet. Moreover, FCR of chicks fed high-energy starter diet was superior to those fed low-energy diet [38]. Wang et al. (2014) [37] noted that high nutrient density improves FCR during the grower feeding phase. Additional findings in our study showed that BW and BWG were not significantly influenced by BMD, which is in agreement with a previous study by Damron et al. [39]. On the contrary, BMD improved the BW in broilers [40]. In the current study, we found that BMD significantly improved FCR in chickens on days 25–35. Our result is supported by a previous study that also established that FCR was significantly improved in birds treated with bacitracin [41]. Sims et al. [17] affirmed that at week 18, FCR was significantly lower in chickens fed with BMD than in the control group. Surprisingly, there was no interaction between BMD and energy level on growth performance parameters in our study.

The relative weight of immune organs is one of the primary determining factors of poultry birds’ immune status [42]. Our findings indicated no significant differences among the treatments in the relative weight of organs except for bursa weight, which was significantly increased (*p* = 0.0220) among the chicken birds that received high-energy diets compared to control. The bursa of Fabricius (BF) has been studied and revealed as a primary lymphoid organ in birds; it plays a vital role in the differentiation of B lymphocytes for immunoglobulin production during an immune challenge [43] and the development of the adaptive immune response of birds [44]. Conflicting with our findings, Cho et al. [45] concluded that emulsifier and multienzyme in low-density diets could increase the relative bursa weight. A recent study reported that a low-energy-density diet could increase chicken bursa weight [46]. High-energy diet could also be associated with the roles of the bursa in poultry birds. Chickens fed with a high-energy diet with BMD had significantly increased villus height and width in the current study. This observation indicates that BMD may be more effective in improving intestinal health when a high-energy diet is fed to chickens compared to when a normal-energy diet is fed. Adebowale et al. [47] reported that high dietary energy density induced stress in piglets and reduced villus height/crypt depth ratio in the ileum and duodenum. This indicates that a high-energy diet could be stressful to the chickens’ intestinal functionality. In the current study, the negative effect of feeding high-energy diet on the intestinal structure was ameliorated by the administration of BMD to the chickens. This agrees with previous studies that reported that low doses of BMD increased villus height through the small intestine in chickens [48]. The increase in villus height and surface area indicates an increase in cell proliferation, which may contribute to intestinal epithelial integrity maintenance [49]. 

### 4.2. Effect of BMD and Energy Density on Gut Microbiota Dynamics

Diets and antibiotics have been reported to shape the composition of gut microbiota [50]. Antibiotics sometimes have continuing effects on microbial flora in the gut, leading to a decrease in the abundance of beneficial microbes and promoting harmful bacteria [51]. Contrarily, a study revealed that the addition of BMD or *Bacillus subtilis* significantly reduced the population of harmful bacteria in the intestine [40]. Dietary components are vital for poultry gut health [52]. We investigated the effects of BMD on chicken gut microbiota at different dietary energy levels. The most abundant phyla in broiler chickens were Firmicutes and Bacteroidota, which is consistent with the results of our previous study [7]. Firmicutes produce molecules directly absorbed by the host gut wall as an energy source, and their abundance has been linked to weight gain in chickens [53,54]. It is worth noting that the inclusion of BMD into HE diets improved the population of Firmicutes slightly, compared to the control (HE-BAS), in this current study. Additionally, the abundance of Bacteroidota in broiler chickens that we observed in chickens fed a high-energy diet could result from this microbe’s involvement in the fermentation of dietary carbohydrates to produce short-chain fatty acids (SCFAs) [55]. SCFAs are the main end products of fermentation of nondigestible carbohydrates that colonize the human gut; they serve as sources of energy and have lasting impacts on host physiology [56].

We observed a slight reduction in the relative abundance of intestinal bacteria in the BMD treatment group, which is consistent with our previous findings [7]. The reduction in the relative abundance of *Bacteroidetes* observed among chickens fed with BMD agrees with a recent study that reported that antibiotic treatment reduced *Bacteroidetes* diversity in infants [57]. *Bacteroidetes* members are essential in developing a stable and healthy gut microbiota [58]. Moreover, the BMD effect was seen to significantly decrease *Streptococcus* abundance in our study. *Streptococcus* regulates the production of bacteriocin through quorum sensing [59]. Recently, the leading causative agent of dental caries in humans has been linked with *Streptococcus* mutants [60]. Further research is required to establish the exact role of *Streptococcus* in chickens. 

We detected significant differences in microbial abundance among dietary treatment groups using STAMP software. *Oscillopirales_ge* abundance was significantly increased in chickens fed with high- and normal-energy diets containing BMD compared to those fed with high-energy basal diet. This suggests that BMD may contribute to indigestion in chickens. A high abundance of *Oscillopirales* may play an increasing role in constipation [61]. A significant increase in *Streptococcus* in chickens fed a normal-energy basal diet inhibited the growth of *Oscillospirales*. In contrast, a high-energy diet with BMD drastically reduced the abundance of *Streptococcus*. This phenomenon supports a previous study that established negative correlations between *Streptococcus* and some bacteria. *Streptococcus* has been found to produce metabolites that work against or kill some other bacteria [62,63], such as *Oscillospirales*. Meanwhile, the abundance of some microbes, such as *Faecalibacterium*, was not affected by diet but changed dynamically with time.

One of the essential factors that influences gut bacterial composition is the birds’ age [64]. We examined how the gut microbiota changes dynamically with growth stages and found that *Faecalibacterium* decreased as chickens’ age increased in our study, which is inconsistent with the findings of Donaldson et al. [65], which recorded an increase in the relative abundance of *Faecalibacterium* with time, though in slow proportion. The enrichment of *Faecalibacterium* is associated with a healthy human adult [65] and might influence chicken immune development [66]. The variation in the two studies might be due to diet or antibiotic treatment, which has been described to alter gut microbiota’s composition despite its stability [65]. The proportion of *Alistipes* increased as broiler chickens increased with age in the present study. *Alistipes* are commensal bacteria that belong to the phylum Bacteroidata and are assumed to benefit the host gut [67]. We further observed significant microbes at different time points of our study (days 21, 36, and 43). *Ruminococcus* was significantly abundant on day 36 of our study. Previous studies have reported that *Ruminococcus* might play a role in increasing mucin production by goblet cells [68] and improving host resistance to harmful bacterial invasion in calves [69]. The high abundance of *Bacteroides* and *Ruminococcus* suggests a healthy gut microbiota [70]. *Lactobacillus* was significantly abundant on day 43 based on the LEfSe result in our study. Increased *Lactobacillus* abundance could help inhibit some pathogens due to the production of vitamins and organic acids by this beneficial probiotic [71,72]. In the current study, the decrease in *Lactobacillus* abundance resulting from BMD inclusion signifies an adverse effect of antibiotics on beneficial microbes. A decline in *Lactobacillus* abundance is consistent with an earlier study that reported that antimicrobial growth promoters were associated with a reduction in the abundance of *Lactobacillus* species [73]. An upsurge of lactobacilli may be related to broiler growth depression in relation to competing for nutrient uptake or fat absorption in broiler chickens [10].

Our results showed that there were some interactions between diets and age, based on post hoc pairwise test, that influence the microbial community of the broiler chickens. This finding is consistent with a previous study that has established that the chicken gut microbiota is mostly shaped by the age and diet of the birds [64].

## 5. Conclusions

In conclusion, dietary supplementation of BMD in a high-energy diet improved FCR during the grow-out phase, increased villus height and width, and increased the relative abundance of beneficial gut microbiota members such as Firmicutes in broiler chickens. This study is novel because, to our knowledge, it is the first to present the effects of BMD in a high-energy-density diet on growth performance, organ weights, jejunal morphology, and gut microbiota of broiler chickens. Our results imply that BMD may be more effective in improving intestinal health when supplemented in a high-energy diet for broiler chickens. It also affirmed that chicken gut microbiota is mostly shaped by age and diet. 

## Figures and Tables

**Table 1:** Ingredient, calculated, and analyzed compositions of the basal diets used in the study.

	Starter		Grower		Finisher	
	Normal Energy	High Energy	Normal Energy	High Energy	Normal Energy	High Energy
Ingredient composition						
Corn	41.33	42.66	44.32	39.68	48.48	44.11
Soybean meal (46.5% CP)	40.17	36.43	36.48	38.70	31.52	33.52
Wheat	10.00	10.00	10.00	10.00	10.00	10.00
Vegetable Oil	3.43	5.87	4.59	7.00	5.67	8.04
Limestone	1.80	1.80	1.65	1.64	1.52	1.51
Dicalcium phosphate	1.23	1.21	1.06	1.05	0.93	0.92
Pellet Binding Agent ^Y^	0.50	0.50	0.50	0.50	0.50	0.50
DL-Methionine Premix ^X^	0.61	0.63	0.53	0.56	0.49	0.52
Vitamin–Mineral Premix ^W,V^	0.50	0.50	0.50	0.50	0.50	0.50
Salt	0.40	0.40	0.37	0.37	0.38	0.38
HCL Lys	0.03	0.00	0.00	0.00	0.01	0.00
Calculated composition						
Folic acid (ppm)	2.20	2.20	2.20	2.20	2.20	2.20
Digestible Trp	0.25	0.26	0.23	0.24	0.21	0.22
Digestible Thr	0.87	0.90	0.82	0.84	0.74	0.76
Digestible Met + Cys	0.95	0.98	0.87	0.90	0.80	0.83
Digestible Lys	1.28	1.32	1.16	1.21	1.03	1.07
ME, kcal/kg	3000	3100	3100	3200	3200	3300
Crude protein	23.0	23.8	21.5	22.2	19.5	20.1
Calcium	0.96	0.96	0.87	0.87	0.79	0.79
Available P	0.48	0.48	0.44	0.44	0.40	0.40
Sodium	0.19	0.19	0.18	0.18	0.18	0.18
ME/CP	130	130	144	144	164	164
ME/Digestible Lys	2344	2349	2672	2645	3107	3084
ME/Met + Cys	3158	3163	3563	3556	4000	3976
ME/Digestible Thr	3448	3444	3781	3810	4324	4342
ME/Trp	12,000	11,923	13,478	13,333	15,238	15,000
Analyzed composition (%, except where otherwise stated)						
ME, kcal/kg	3022	3145	3109	3203	3220	3317
Crude protein	24.2	25.2	21.7	22.9	20.2	20.3
Crude fat	6.11	8.03	6.41	8.69	7.04	6.70
Dry matter	88.8	89.1	86.2	87.9	87.7	86.7

^X^ Supplied/kg premix: DL-methionine, 0.5 kg; wheat middlings, 0.5 kg. ^Y^ Pel-stik: Uniscope, Inc., Johnstown, CO, USA. ^W^ Starter vitamin–mineral premix contained the following per kg of diet: 9750 IU vitamin A, 2000 IU vitamin D3, 25 IU vitamin E, 2.97 mg vitamin K, 7.6 mg riboflavin, 13.5 mg Dl Ca-pantothenate, 0.012 mg vitamin B12, 29.7 mg niacin, 1.0 mg folic acid, 801 mg choline, 0.3 mg biotin, 4.9 mg pyridoxine, 2.9 mg thiamine, 70.2 mg manganese, 80.0 mg zinc, 25 mg copper, 0.15 mg selenium, 50 mg ethoxyquin, 1543 mg wheat middlings, and 500 mg ground limestone. ^V^ Grower and Finisher vitamin–mineral premix contained the following per kg of diet: 9750 IU vitamin A, 2000 IU vitamin D3, 25 IU vitamin E, 2.97 mg vitamin K, 7.6 mg riboflavin, 13.5 mg Dl Ca-pantothenate, 0.012 mg vitamin B12, 29.7 mg niacin, 1.0 mg folic acid, 801 mg choline, 0.3 mg biotin, 4.9 mg pyridoxine, 2.9 mg thiamine, 70.2 mg manganese, 80.0 mg zinc, 25 mg copper, 0.15 mg selenium, 50 mg ethoxyquin, 1543 mg wheat middlings, and 500 mg ground limestone.

**Table 2:** Effect of dietary energy density and bacitracin withdrawal on growth performance of broiler chickens.

	Normal Energy		High Energy		SEM ^1^	*p*-Value		
	Basal	BMD ^2^	Basal	BMD		BMD	Energy	BMD × Energy
Feed intake, g/bird								
Days 0–7	164	164	167	171	5.07	0.670	0.363	0.724
Days 8–14	354	362	350	351	9.91	0.636	0.448	0.727
Days 15–24	1129	1068	1288	1059	97.8	0.149	0.447	0.398
Days 25–35	1837	1795	1824	1753	25.4	0.035	0.279	0.574
Days 35–42	1186	1402	1198	1187	83.8	0.232	0.235	0.186
Days 0–42	4670	4791	4826	4520	137	0.507	0.679	0.130
Body weight, g								
Day 0	43.6	43.3	43.2	43.2	0.29	0.658	0.390	0.658
Day 7	186 ^a^	177 ^ab^	177 ^ab^	171 ^b^	3.70	0.131	0.010	0.892
Day 14	501	492	502	506	13.5	0.830	0.573	0.611
Day 24	1310	1290	1284	1287	13.6	0.517	0.289	0.404
Day 35	2471	2453	2457	2477	23.0	0.966	0.829	0.433
Day 42	3233	3247	3246	3291	38.2	0.447	0.464	0.681
Body weight gain, g/bird								
Days 0–7	142 ^a^	138 ^ab^	134 ^ab^	127 ^b^	3.73	0.151	0.012	0.842
Days 8–14	317	314	326	332	13.2	0.929	0.332	0.733
Days 15–24	807	795	781	785	15.9	0.789	0.276	0.639
Days 25–35	1161	1164	1173	1190	15.9	0.545	0.228	0.675
Days 25–42	762	794	789	814	22.0	0.204	0.297	0.902
Days 0–42	3190	3204	3203	3248	38.2	0.446	0.455	0.687
Feed conversion ratio								
Days 0–7	1.15 ^b^	1.19 ^ab^	1.25 ^ab^	1.38 ^a^	0.06	0.194	0.017	0.609
Days 8–14	1.13	1.16	1.09	1.07	0.05	0.871	0.173	0.663
Days 15–24	1.41	1.34	1.67	1.35	0.13	0.135	0.360	0.414
Days 25–35	1.59 ^a^	1.54 ^a^	1.56 ^a^	1.47 ^b^	0.02	0.003	0.009	0.286
Days 25–42	1.55	1.80	1.52	1.46	0.12	0.667	0.117	0.220
Days 0–42	1.47	1.50	1.51	1.39	0.01	0.335	0.378	0.099

In a row, means assigned different lowercase letters are significantly different, *p* < 0.05 (Tukey’s procedure). ^1^ Standard error of the mean. ^2^ Bacitracin methylene disalicylate.

**Table 3:** Effect of dietary energy density and BMD on organ weights (g/kg body weight).

Parameter	Normal Energy		High Energy		SEM ^1^	*p*-Value		
	Basal	BMD ^2^	Basal	BMD		BMD	Energy	BMD × Energy
Spleen	0.74	0.86	0.83	0.76	0.07	0.738	0.890	0.148
Ceca	5.70	4.55	4.61	4.60	0.51	0.260	0.314	0.264
Liver	15.8	15.5	15.4	15.0	0.53	0.484	0.417	0.883
Bursa	1.48	1.28	1.65	1.72	0.14	0.608	0.022	0.281
Heart	4.80	5.20	4.80	4.99	0.21	0.175	0.609	0.622

^1^ Standard error of the mean. ^2^ Bacitracin methylene disalicylate.

**Table 4:** Effect of dietary energy level and BMD on jejunal morphology (mm) of broiler chickens.

Parameter	Normal Energy		High Energy		SEM ^1^	*p*-Value		
	Basal	BMD ^2^	Basal	BMD		BMD	Energy	BMD × Energy
Villus height	1.51 ^ab^	1.33 ^b^	1.51 ^ab^	1.63 ^a^	0.04	0.630	0.019	0.051
Villus width	0.18 ^a^	0.19 ^a^	0.12 ^b^	0.19 ^a^	0.01	0.007	0.519	0.017
Crypt depth	0.17	0.18	0.15	0.17	0.01	0.179	0.552	0.666
VH/CD ^3^	9.72	7.78	9.63	9.36	0.39	0.121	0.062	0.453

In a row, means assigned different lowercase letters are significantly different, *p* < 0.05 (Tukey’s procedure). ^1^ Standard error of the mean; ^2^ Bacitracin methylene disalicylate. ^3^ Villus height/crypt depth ratio.

**Table 5:** Fifteen OTUs that were differentially abundant amongst treatments (NE-BAS, NE-BMD, HE-BAS, and HE-BMD) (FDR < 0.05).

	log_2_ Fold Change	*p*-Value	Padj	Phylum	Genus
Otu00013	10.27628	3.77 × 10^−104^	3.76 × 10^−101^	Bacteroidota	*Bacteroidia_unclassified*
Otu00008	12.16469	1.10 × 10^−52^	5.50 × 10^−50^	Bacteroidota	*Alistipes*
Otu00210	6.015742	2.53 × 10^−34^	8.40 × 10^−32^	Bacteroidota	*Bacteroidia_unclassified*
Otu00016	12.06219	1.26 × 10^−29^	3.15 × 10^−27^	Firmicutes	*Peptostreptococcaceae_unclassified*
Otu00081	−5.50531	5.47 × 10^−23^	1.09 × 10^−20^	Firmicutes	*Faecalibacterium*
Otu00004	12.52144	6.05 × 10^−21^	1.00 × 10^−18^	Bacteroidota	*Bacteroides*
Otu00309	6.301178	1.10 × 10^−20^	1.57 × 10^−18^	Firmicutes	*Ruminococcus*
Otu00209	5.807156	1.99 × 10^−19^	2.47 × 10^−17^	Bacteroidota	*Alistipes*
Otu00001	−3.82554	4.49 × 10^−19^	4.96 × 10^−17^	Firmicutes	*Faecalibacterium*
Otu00039	6.494405	2.03 × 10^−16^	2.02 × 10^−14^	Bacteroidota	*Bacteroides*
Otu00280	−5.7675	2.99 × 10^−16^	2.70 × 10^−14^	Firmicutes	*Faecalibacterium*
Otu00071	10.03166	1.45 × 10^−15^	1.21 × 10^−13^	Firmicutes	*Bacillales_unclassified*
Otu00079	8.499449	1.78 × 10^−15^	1.36 × 10^−13^	Firmicutes	*Lachnospiraceae_unclassified*
Otu00229	6.941029	1.93 × 10^−15^	1.37 × 10^−13^	Firmicutes	*Firmicutes_unclassified*
Otu00150	3.574264	2.30 × 10^−15^	1.53 × 10^−13^	Firmicutes	*Lachnospiraceae_unclassified*

## Data Availability

Not applicable.

## Supplementary Materials

The following are available online at https://www.mdpi.com/article/10.3390/microorganisms9040787/s1, Figure S1: Sequences quality for R1; Figure S2: Sequences quality for R2; Table S1: Beta-diversity among sampling factors results from PERMANOVA; Table S2: Post-Hoc Pairwise test contrasts for multiple comparisons.
