# Supplementary material for: Gut Microbiota Dynamics, Growth Performance, and Gut Morphology in Broiler Chickens Fed Diets Varying in Energy Density with or without Bacitracin Methylene Disalicylate (BMD)
Source: Microorganisms. 2021 Apr 9;9(4):787. doi: 10.3390/microorganisms9040787 (PMC8070028; doi:10.3390/microorganisms9040787)
Supplement: Supplementary file 1 [file microorganisms-09-00787-s001.pdf]

Supplementary Materials

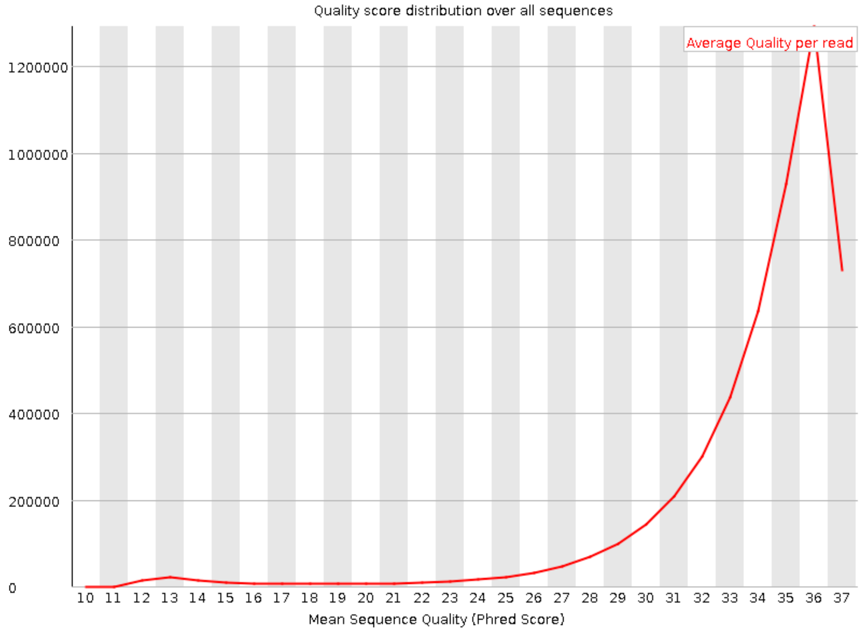

Figure S1 Sequences quality for R1

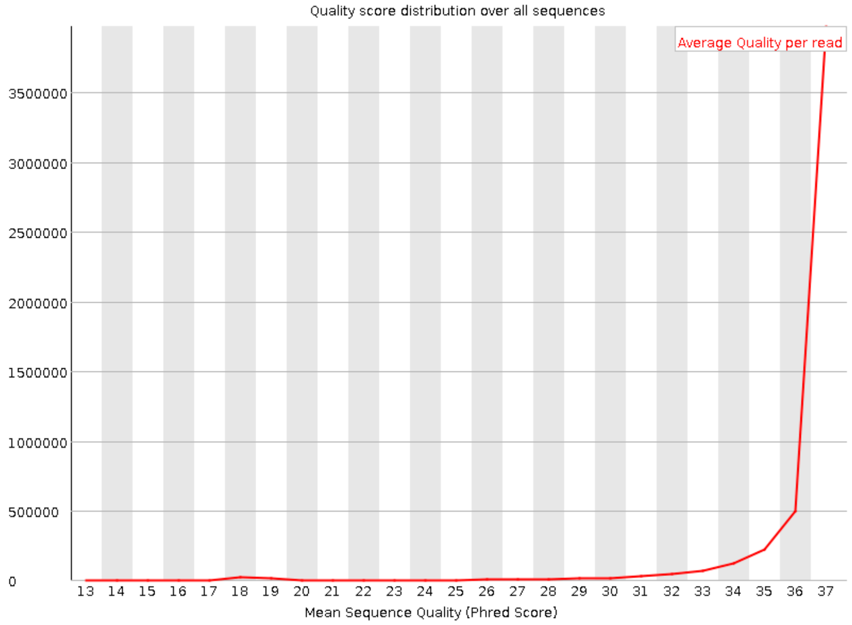

Figure S2 Sequences quality for R2

Table S1 Beta-diversity among sampling factors results from PERMANOVA

Sampling factor	R2	Pr(>F)
Energy.level	0.0055	0.8435
Antibiotic	0.0166	0.0679
Diets	0.0068	0.6754
Sampling	0.2552	0.0001
Residuals	0.7159	NA
Total	1.0000	NA

Table S2 Post-Hoc Pairwise test contrasts for multiple comparisons.

**pairs**	**R2**	**P. adjusted**
Diet1.Day 21 vs Diet6.Day 21	0.083	0.318
Diet1.Day 21 vs Diet2.Day 21	0.045	0.945
Diet1.Day 21 vs Diet7.Day 21	0.072	0.700
Diet1.Day 21 vs Diet1.Day 36	0.258	0.007
Diet1.Day 21 vs Diet2.Day 36	0.286	0.004
Diet1.Day 21 vs Diet6.Day 36	0.235	0.004
Diet1.Day 21 vs Diet7.Day 36	0.181	0.008
Diet1.Day 21 vs Diet1.Day 43	0.317	0.004
Diet1.Day 21 vs Diet2.Day 43	0.278	0.004

pairs	R2	P. adjusted
Diet1.Day 21 vs Diet6.Day 43	0.330	0.004
Diet1.Day 21 vs Diet7.Day 43	0.316	0.004
Diet6.Day 21 vs Diet2.Day 21	0.085	0.401
Diet6.Day 21 vs Diet7.Day 21	0.088	0.476
Diet6.Day 21 vs Diet1.Day 36	0.370	0.004
Diet6.Day 21 vs Diet2.Day 36	0.389	0.004
Diet6.Day 21 vs Diet6.Day 36	0.336	0.004
Diet6.Day 21 vs Diet7.Day 36	0.285	0.008
Diet6.Day 21 vs Diet1.Day 43	0.444	0.007
Diet6.Day 21 vs Diet2.Day 43	0.417	0.008
Diet6.Day 21 vs Diet6.Day 43	0.468	0.004
Diet6.Day 21 vs Diet7.Day 43	0.453	0.004
Diet2.Day 21 vs Diet7.Day 21	0.053	0.932
Diet2.Day 21 vs Diet1.Day 36	0.276	0.004
Diet2.Day 21 vs Diet2.Day 36	0.273	0.013
Diet2.Day 21 vs Diet6.Day 36	0.247	0.004
Diet2.Day 21 vs Diet7.Day 36	0.190	0.004
Diet2.Day 21 vs Diet1.Day 43	0.336	0.007
Diet2.Day 21 vs Diet2.Day 43	0.301	0.007
Diet2.Day 21 vs Diet6.Day 43	0.361	0.008
Diet2.Day 21 vs Diet7.Day 43	0.333	0.008

pairs	R2	P. adjusted
Diet7.Day 21 vs Diet1.Day 36	0.270	0.021
Diet7.Day 21 vs Diet2.Day 36	0.279	0.013
Diet7.Day 21 vs Diet6.Day 36	0.245	0.004
Diet7.Day 21 vs Diet7.Day 36	0.197	0.033
Diet7.Day 21 vs Diet1.Day 43	0.354	0.012
Diet7.Day 21 vs Diet2.Day 43	0.320	0.010
Diet7.Day 21 vs Diet6.Day 43	0.373	0.008
Diet7.Day 21 vs Diet7.Day 43	0.352	0.008
Diet1.Day 36 vs Diet2.Day 36	0.131	0.199
Diet1.Day 36 vs Diet6.Day 36	0.049	0.800
Diet1.Day 36 vs Diet7.Day 36	0.066	0.631
Diet1.Day 36 vs Diet1.Day 43	0.092	0.509
Diet1.Day 36 vs Diet2.Day 43	0.131	0.080
Diet1.Day 36 vs Diet6.Day 43	0.078	0.569
Diet1.Day 36 vs Diet7.Day 43	0.121	0.199
Diet2.Day 36 vs Diet6.Day 36	0.066	0.597
Diet2.Day 36 vs Diet7.Day 36	0.081	0.463
Diet2.Day 36 vs Diet1.Day 43	0.161	0.066
Diet2.Day 36 vs Diet2.Day 43	0.142	0.064
Diet2.Day 36 vs Diet6.Day 43	0.173	0.044
Diet2.Day 36 vs Diet7.Day 43	0.114	0.199

pairs	R2	P. adjusted
Diet6.Day 36 vs Diet7.Day 36	0.056	0.696
Diet6.Day 36 vs Diet1.Day 43	0.104	0.231
Diet6.Day 36 vs Diet2.Day 43	0.125	0.049
Diet6.Day 36 vs Diet6.Day 43	0.106	0.199
Diet6.Day 36 vs Diet7.Day 43	0.108	0.163
Diet7.Day 36 vs Diet1.Day 43	0.127	0.199
Diet7.Day 36 vs Diet2.Day 43	0.112	0.106
Diet7.Day 36 vs Diet6.Day 43	0.120	0.137
Diet7.Day 36 vs Diet7.Day 43	0.107	0.242
Diet1.Day 43 vs Diet2.Day 43	0.082	0.440
Diet1.Day 43 vs Diet6.Day 43	0.052	0.886
Diet1.Day 43 vs Diet7.Day 43	0.065	0.692
Diet2.Day 43 vs Diet6.Day 43	0.090	0.249
Diet2.Day 43 vs Diet7.Day 43	0.070	0.503
Diet6.Day 43 vs Diet7.Day 43	0.077	0.503
